# Arthroscopic reconstruction of the medial patellofemoral ligament in skeletally immature patients using the modified sling procedure: a novel technique for MPFL reconstruction

**DOI:** 10.1186/s13018-023-03775-9

**Published:** 2023-05-05

**Authors:** Qingguo Zhang, Liwei Ying, Dawei Han, Lingchao Ye, Tao-Hsin Tung, Junbo Liang, Peihong Liu, Xiaobo Zhou

**Affiliations:** 1 Department of Sports Medicine, Taizhou Hospital of Zhejiang Province, Wenzhou Medical University, Linhai, Zhejiang China; 2 Evidence-Based Medicine Center, Public Laboratory, Taizhou Hospital of Zhejiang Province, Wenzhou Medical University, Linhai, Zhejiang China

**Keywords:** Medial patellofemoral ligament, Reconstruction, Arthroscopy, Patellofemoral instability, Peroneus longus tendon, Adductor magnus

## Abstract

**Background:**

Patellar dislocation is common in young people. Although isolated anatomic double-bundle reconstruction of the MPFL is a common and effective surgical treatment for patellofemoral instability, concerns about the risk of injury to the epiphysis remain.

**Methods:**

A total of 21 children and adolescents (9 males, 12 females; mean age: 10.7 years; range: 8 to 13 years) with recurrent patella dislocation or symptomatic instability following a primary dislocation were enrolled in the study. In all patients, double-bundle medial patellofemoral ligament (MPFL) reconstruction and femoral sling procedure were performed under arthroscopy, using an anterior half peroneus longus tendon (AHPLT) autograft. Functional outcomes were evaluated preoperatively and during follow-ups based on Kujala and Lysholm scores. Radiological examinations including radiographs, 3D-CT, and MRI were performed pre- and post-operatively.

**Results:**

Among two-year postoperative follow-up (range: 24–42 months) showed significant improvement in functional scores (*p* < 0.01). The Lysholm score increased from 68 (44.5) to 100 (0) and the Kujala score increased from 26 (34.5) to 100 (2) The patellar tilt angel improved significantly (*p* < 0.01) from 24.3° ± 10.4 preoperatively to 11.9° ± 7.0 postoperatively. MRIs performed 6- and 12-months post operation did not show any signs of dysfunction of the reconstructed MPFL or cartilage degeneration.

**Study design:**

Case Series; Level of evidence, 4.

**Conclusion:**

Arthroscopic reconstruction of the MPFL using the modified sling procedure is an effective procedure for the treatment of patellar instability in skeletally immature patients.

## Introduction

The medial patellofemoral ligament (MPFL) is the main medial static stabilizer that restrains patellar lateralization during the initial 30° of knee flexion prior to patellar engagement into trochlea groove [1]. Rupture of the MPFL occurs in over 90% of all first patella dislocations and approximately 100% of re-dislocations [2]. Isolated MPFL reconstruction, especially anatomic double-bundle reconstruction, is the most common and effective surgical treatment for patellar instability in pediatric patients without severe osseous deformity [3–5]. However, femoral insertion of the MPFL is near to the distal femoral physis in children and adolescents [6]. The femoral drilling in the MPFL insertion is safe when performed properly but technically challenging [7, 8].

**Scheme 1.**
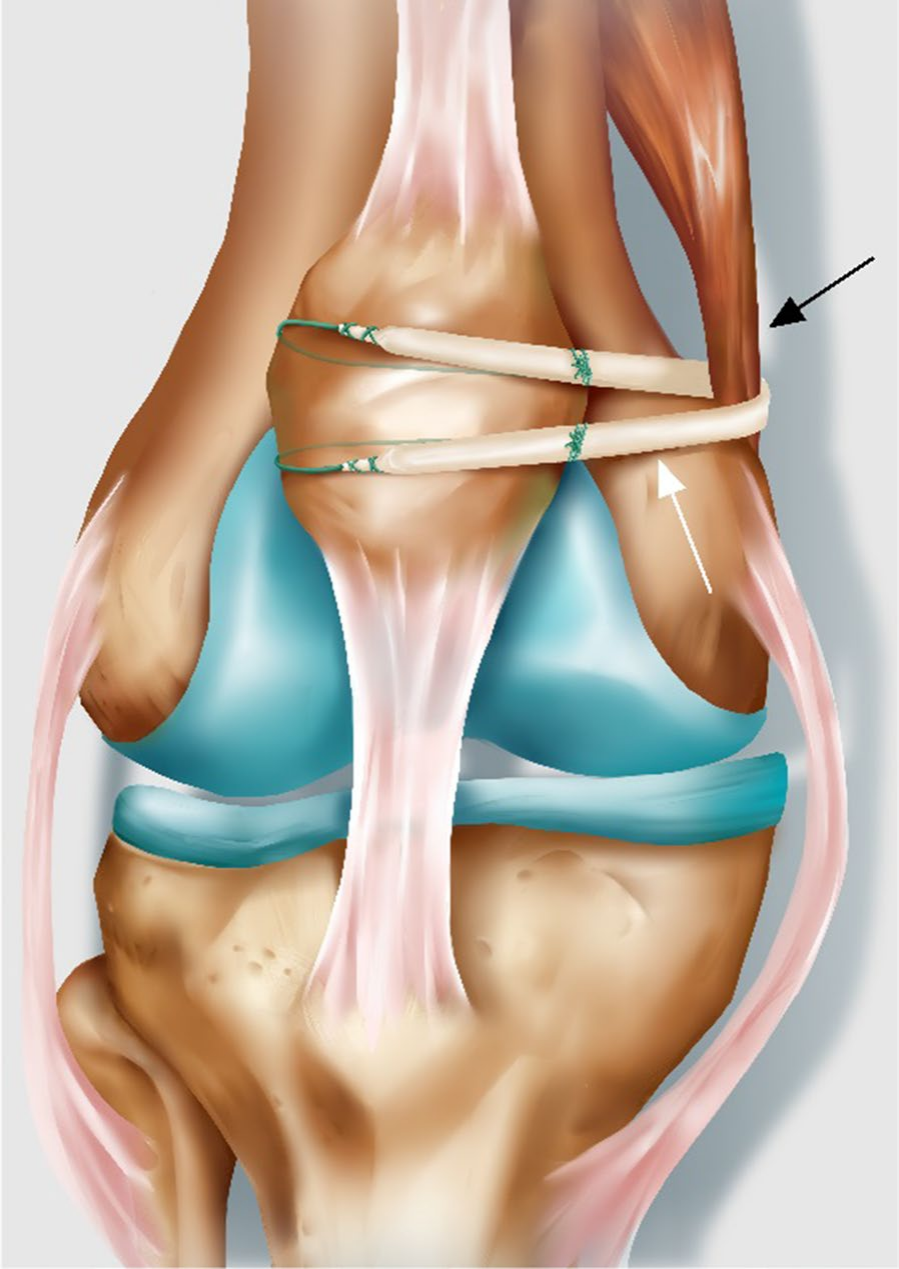
Postoperative schematic diagram of knee joint. White arrow: anterior half peroneus longus tendon autografts; black arrow: adductor magnus tendon

To avoid drilling at the femoral insertion and preserve the epiphysis, several MPFL reconstruction techniques, such as pedicled adductor transplantation, adductor sling technique, and medial collateral ligament (MCL) sling, have been proposed [9, 10]. However, the clinical outcomes of these techniques in children and adolescents have not been sufficiently investigated. In this study, we conducted anatomic double-bundle reconstruction in skeletally immature patients using a modified sling procedure (Scheme 1) under arthroscopy to minimize the risk of epiphyseal injury. The aim of this study was to evaluate outcomes of the modified adductor tendon sling procedure in children and adolescents with patellofemoral instability.

## Methods

This retrospective study enrolled 21 patients with open physes who underwent double-bundle MPFL reconstruction under arthroscopy using anterior half peroneus longus tendon (AHPLT) autograft and femoral sling procedure between 2018 and 2020 (Table 1). The study was approved by the Medical Ethics Committee of Taizhou Hospital Affiliated to Wenzhou Medical University (K20211225). Children and adolescents with recurrent patella dislocation or symptomatic instability following primary dislocation were enrolled for the study. The exclusion criteria were as follows: fractures requiring open reduction or fixation and ipsilateral anterior/posterior cruciate ligament injury. Elevated tibial tuberosity trochlear groove distance (TT-TG > 2.0) and patella alta (Caton-Deschamps > 1.2) were no contraindication.

**Table 1:** Pre-operative information and parameters about the cohort

Patients	Male (M)/Female (F)	Left (L)/ Right (R)	Age (Y)	Times of dislocations	Previous surgery	Trochlear dysplasia (Dejour)	Patella alta (CD)	TT-TG	Patellar (P)/ condyle (C) fracture
Patient 1	M	L	10	1	None	B	1.2	1.4	P
Patient 2	M	L	9	2	None	D	1.08	0.82	None
Patient 3	M	R	9	2	None	B	0.94	1.62	None
Patient 4	M	L	10	1	None	A	1.41	0.43	None
Patient 5	F	L	11	2	None	D	1.13	1.24	None
Patient 6	F	R	9	1	None	C	0.89	2.05	P
Patient 7	F	R	10	3	None	B	0.78	1.46	None
Patient 8	M	R	12	2	None	B	1.19	1.5	None
Patient 9	F	L	11	2	None	B	1.36	1.24	P
Patient 10	M	L	9	2	None	B	1.26	0.78	None
Patient 11	M	R	10	1	None	B	1.13	2.05	P
Patient 12	F	L	8	1	None	Normal	1.26	0.75	P
Patient 13	F	R	11	2	None	A	1.07	0.73	P
Patient 14	F	R	13	5	None	C	1.02	1.58	P
Patient 15	M	R	13	3	None	D	1.18	0.64	P
Patient 16	F	L	9	2	None	C	0.93	0.82	None
Patient 17	F	R	13	3	None	B	1.32	1.63	None
Patient 18	F	L	11	1	None	B	1.03	1.55	C
Patient 19	F	L	12	1	None	B	1.01	1.38	None
Patient 20	M	R	11	2	None	B	1.1	1.38	P
Patient 21	F	R	13	1	None	C	0.88	1.77	P

**Fig. 1:**
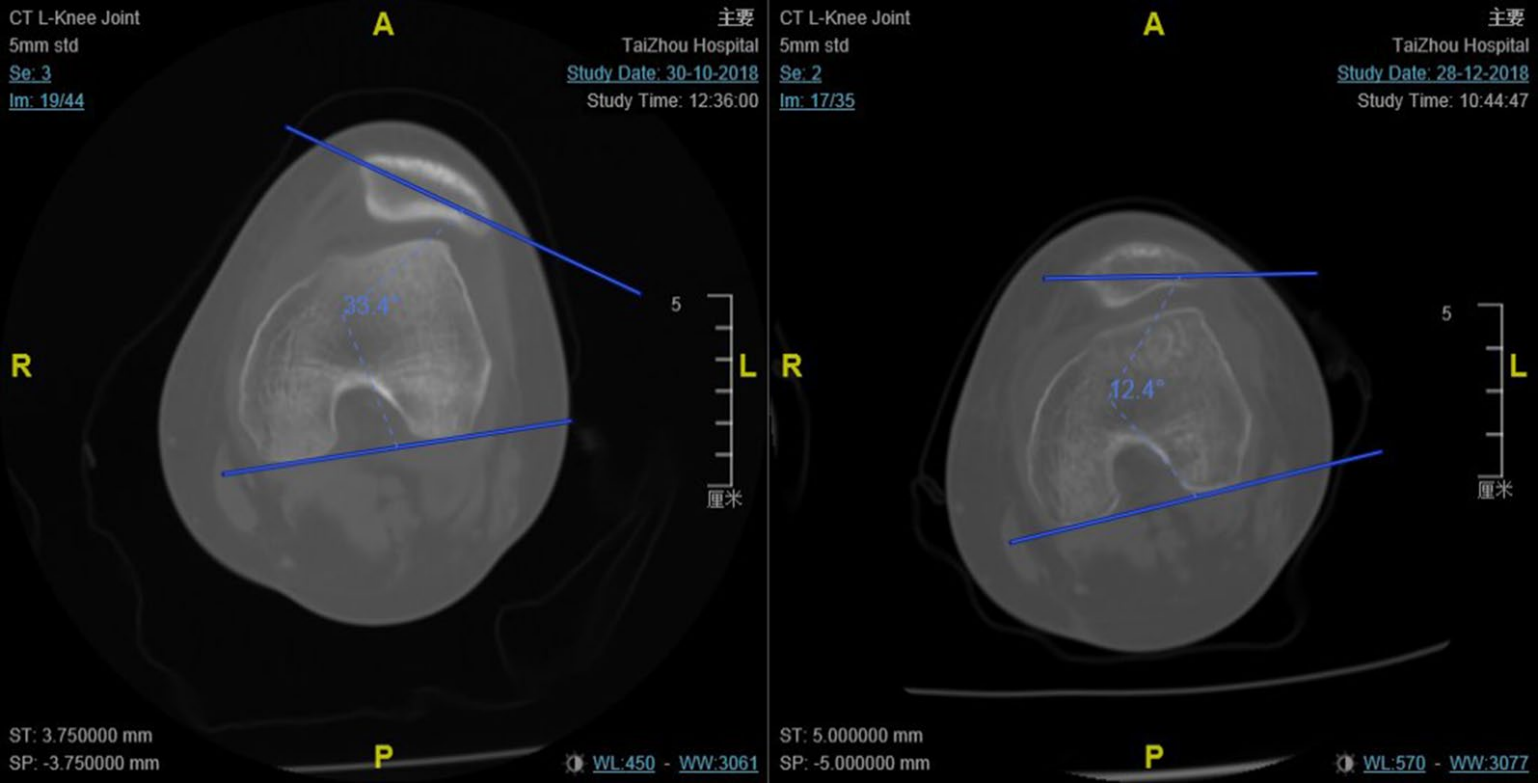
Preoperative and postoperative computerized tomography (CT) scans of the patellar tilt angle (the angle between the femoral posterior condylar line and the line defining the maximal patellar width)

The functional outcomes preoperatively and in subsequent follow-ups (6, 12, 24 months post-operation) were evaluated based on Kujala and Lysholm scores. Further, radiological examination, including preoperative and postoperative radiographs, 3D-CT, and MRI, were conducted. The main evaluation parameter was the patellar tilt angle (the angle between the femoral posterior condylar line and the line defining the maximal patellar width) (Fig. 1). In addition, fracture, cartilage degeneration, tendon healing, and complications were recorded.

### Surgical technique

**Fig. 2:**
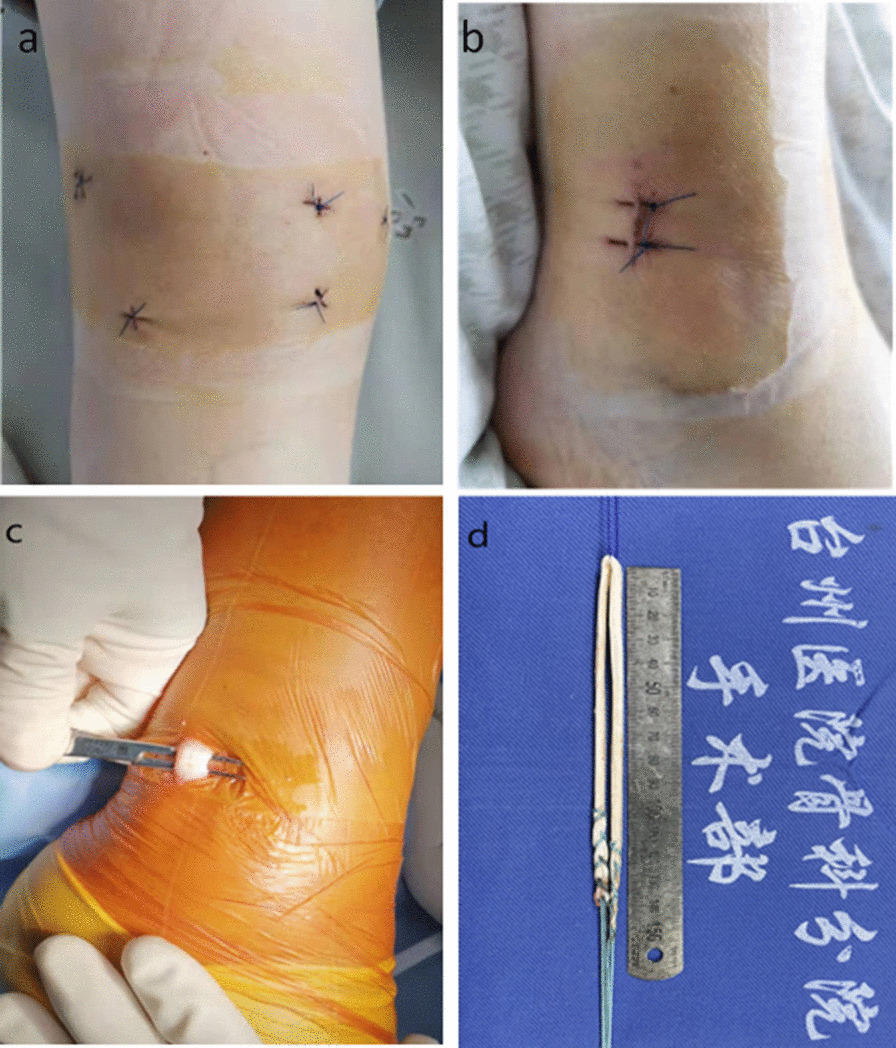
**a** Five approaches distributed around the knee; **b** the incision made for harvesting of the anterior half peroneus longus tendon (AHPLT); **c** the peroneus longus tendon; **d** the braided AHPLT

**Fig. 3:**
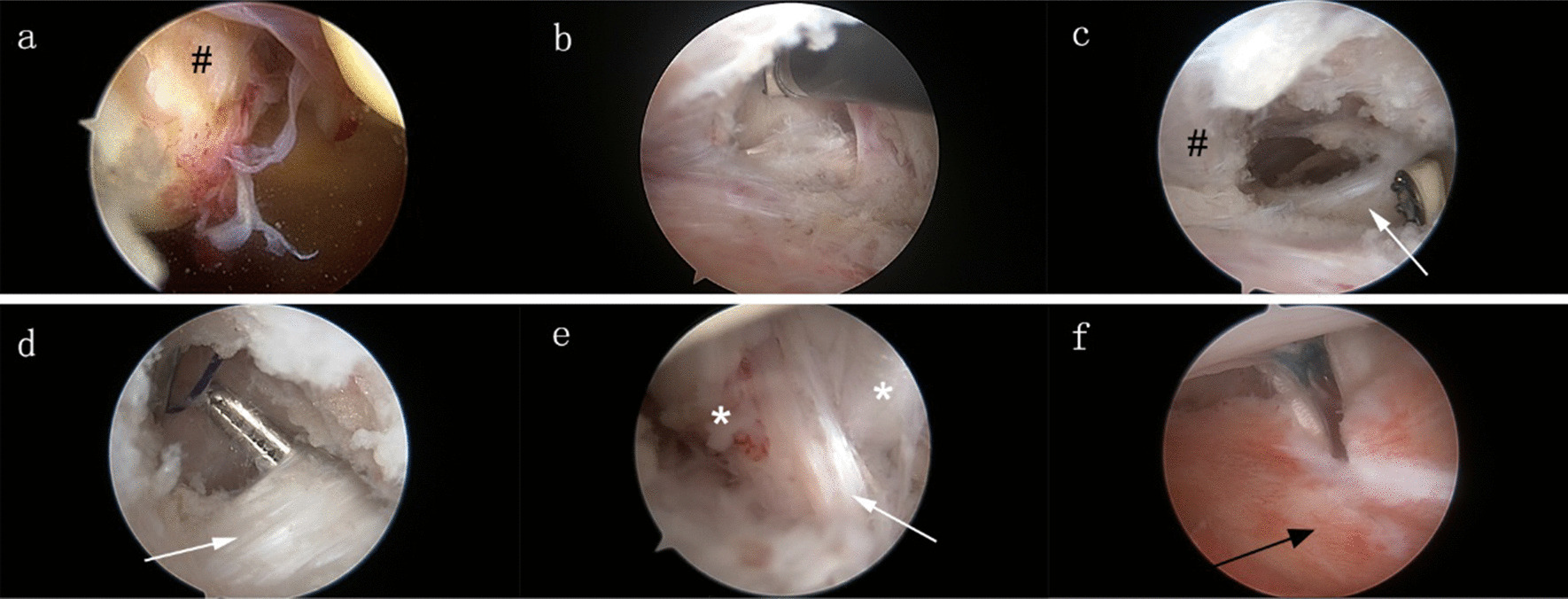
Arthroscopic the medial patellofemoral ligament (MPFL) reconstruction procedure. **a** Tear of the MPFL and the capsule was viewed at the patellar attachment (11 o’clock position); **b** A superomedial capsular window above the synovia plication in the medial gutter was created from inside; **c** The adductor magnus tendon proximal to the MPFL was exposed through the capsular window; **d** The proximal insertion of the adductor magnus was separated by a right-angle clamp and a shuttle suture was threaded; **e** The implanted autograft was introduced under the suture guidance and fixed with a sling around the adductor magnus tendon; **f** The double-bundle autograft was located at the outer layer of the capsule and fastened to the medial facet of the patella. White arrow: adductor magnus; black arrow: capsure; * autograft; # MPFL

Patients were placed on the operation table in a supine position. A physical examination was carried out under anesthesia to evaluate patellar stability before surgery. Standard arthroscopy was performed through anteromedial and anterolateral portals (Fig. 2a) to verify the MPFL injury and other pathologies (Fig. 3a). A 1.5–2 cm longitudinal incision was made over the peroneus tendon behind the lateral ankle (Fig. 2b). The peroneus longus tendon was exposed, and the anterior half of the tendon (1/2 to 2/3) was harvested (Fig. 2c). Subsequently, the graft was trimmed to the required length (14–20 cm), and the ends were braided with No.5 Ethibond sutures (Ethicon, Somerville, NJ, USA) (Fig. 2d).

The arthroscope was moved to the anteromedial approach for observation. Thereafter, auxiliary approaches were established on the medial and lateral sides of the patella and over the medial epicondyle (Fig. 2a). Two 2.0 mm Kirschner wires were used to drill tunnels from the medial upper corner and middle part of the patella to the lateral side. Further, two No. 0 PDSII suture loops were passed through the bone tunnels and reserved as suture shuttles. The posteromedial capsule above the synovia plication in the medial gutter was opened from inside while maintaining knee extension (Fig. 3b). Loose fibrous tissues were debrided to expose the residual MPFL and insertion of the proximal adductor tendon (Fig. 3c). A No.0 PDSII suture was introduced to loop around the adductor magnus tendon (Fig. 3d). Thereafter, the autograft was pulled in (Fig. 3e) under the suture guidance. A vessel clamp was bluntly penetrated from the medial patellar edge to the adductor tuberosity between the second and third tissue layer outside the joint capsule. Both free ends of the autograft were grabbed out and passed across the patella from the patellar aponeurosis surface. Furthermore, the Ethibond sutures of the free ends were folded back through the bone tunnel under the guidance of the reserved PDSII sutures (Scheme 1). The sutures were tensioned and knotted to fasten the tendon to the medial facet of the patella with the knee flexed at 30° (Fig. 3f). To verify appropriate tension of the constructed MPFL, the patella was displaced laterally with the knee flexed at 20°. The range shifted within one quadrant of the patella [11].

### Postoperative rehabilitation

A hinged knee brace was used to protect the injured knee. The patient was encouraged to perform ankle exercise, isometric contraction of the quadriceps. In addition, active extension and passive flexion of knee were encouraged using the contralateral limb. The range of motion was not limited in the non-weight-bearing position. Full weight-bearing walking was allowed with the knee brace locked at full extension in patients who could fully extend the knee joint actively or maintain a straight leg raise for more than one minute. Patients who achieved 90° flexion were discharged from hospital. Full range and full weight-bearing exercises were encouraged under the protection of the brace at three weeks post-surgery. The brace was removed after six weeks. Moreover, the patient was encouraged to perform exercises, such as cycling, jogging, or squatting. They were also allowed participate in recreational sports after three months. After four to six months, patients were allowed to participate in more strenuous sports. Thereafter, they were allowed to engage in competitive sports depending on the muscle strength.

### Statistical analysis

Data analysis was performed with statistical software (SPSS v.25, IBM Inc., Harmonk, NY, USA). Normality of numerical data were assessed by Shapiro–Wilk test. Descriptive statistics were reported as means and standard deviations (SD) for normally distributed numerical data, otherwise medians and interquartile ranges (IQR) were used. Comparisons between groups both at baseline and at follow-up were performed by use of Student’s *t* test for normally distributed data, otherwise the Wilcoxon matched pairs signed rank test was used. Significance was set at *p* ≤ 0.05.

## Results

**Fig. 4:**
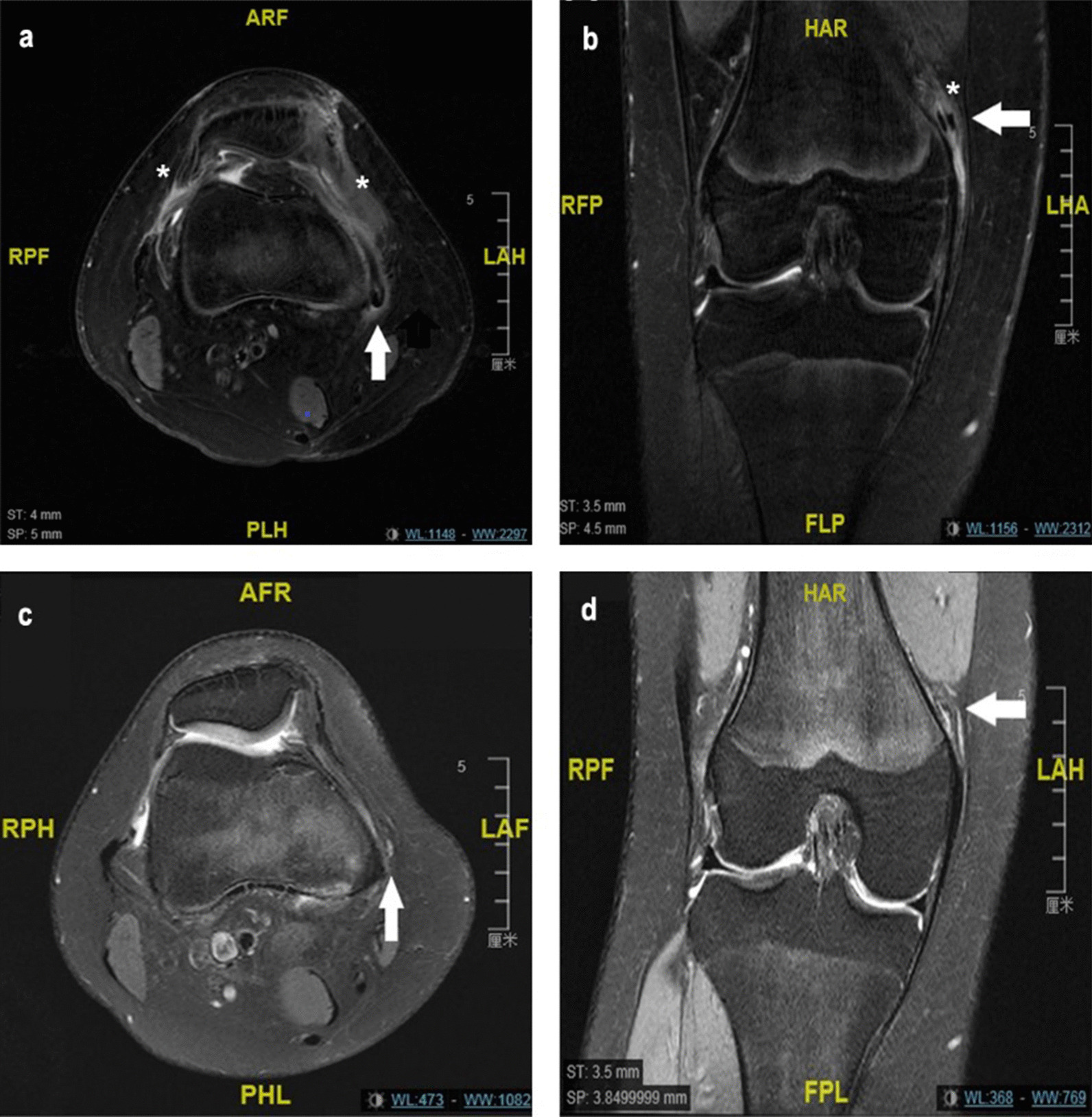
After surgery, patients were followed up every 6 months. The inflammatory response was present postoperatively at 6 months (**a**, **b**), but was disappeared postoperatively at 12 months (**c**, **d**). The reconstructed ligament healed well and no signs of dysfunction were observed in both time points. Relaxation, creep deformation or discontinuity of the reconstructed MPFL was considered to be dysfunction. White arrow: reconstructed MPFL; *: inflammatory response

21 patients (9 males, 12 females; mean age: 10.7 years; range: 8 to 13 years) completed the scheduled follow-up (6, 12, 24 months). The mean age of patients was 10.7 years (range, 8–13) with two years follow-up. Six patellar avulsion fractures and two lateral condylar cartilage exfoliation fractures were recorded during the operation, and debridement was performed. A two-year follow up showed that the functional scores had improved significantly (*p* < 0.01) (Table 2). Lysholm score increased from 68 (44.5) to 100 (0) and the Kujala score increased from 26 (34.5) to 100 (2). There were no recurrent patellar dislocations at the final follow-up. Moreover, the patellar tilt angel improved significantly (*p* < 0.01) from 24.3° ± 10.4 preoperatively to 11.9° ± 7.0 12-months postoperatively (Table 2). Furthermore, no patellar fractures were reported. MRIs of the knee conducted 6 and 12 months postoperatively did not show any signs of dysfunction of the reconstructed MPFL or cartilage degeneration (Fig. 4).

**Table 2:** Results of functional scores and radiological parameters

Variable	Mean ± standard deviations/Median (interquartile ranges)	*p*-value
Lysholm score		*P* < 0.01
Pre-operation	68(44.5)	
12 months post-operation	100(5)	
24 months post-operation	100(0)	
Kujala score		*P* < 0.01
Pre-operation	26(34.5)	
6 months post-operation	93(6)	
12 months post-operation	98(6)	
24 months post-operation	100(2)	
Tilt angle		*P* < 0.01
Pre-operation	24.3 ± 10.4	
12 months post-operation	11.9 ± 7.0

## Discussion

In this study, we conducted MPFL reconstruction in skeletally immature patients using a modified sling procedure under arthroscopy to prevent the risk of epiphyseal injury. Postoperative follow-ups revealed a significant improvement in functional scores and radiological parameters.

Patellar dislocation is common in the young people. Although conservative management has a certain therapeutic effect in patellar dislocation, it is associated with a recurrent rate of 69% [12]. The morphology of patella and femoral trochlear can be improved by maintaining the correct contrapuntal relationship [13–15]. Therefore, early surgical intervention is indicated in skeletally immature patients with primary or recurrent patellar dislocation [16, 17]. Isolated anatomic double-bundle reconstruction of the MPFL is the common and effective surgical treatment for patellofemoral instability [5]. The femoral tunnel point is commonly identified using Schottle’s method [18]. However, in skeletally immature patients, the “Schottle point” is close to the distal femoral physis, which increases risk of injury to the epiphysis [6]. To avoid tunneling on the femoral attachment or hardware placement, non-anatomical reconstruction techniques have been developed. Using adductor magnus insertion as the femoral attachment point of MPFL may show a quasi-isometric effect because the adductor tubercle is close to the superior aspect of the MPFL femoral attachment (the most isometric point) [19]. Additionally, the attachment of MPFL and adductor tendon is a way of elastic fixation, which can compensate for the slight length mismatch of the reconstructed MPFL during mobility [10, 20]. Therefore, the adductor sling procedure with free graft was proposed for the reconstruction of MPFL.

The sling procedure has been described in various studies (Table 3). Gomes et al. showed that the dynamic femoral fixation was superior to that of rigid alternative. Patients who underwent the sling procedure were more likely to feel subjectively better and could participate in sports. The graft was passed through the osteoperiosteal tunnel under the adductor insertion and then looped around the adductor insertion. However, this procedure is associated with a risk of insertion detachment. Furthermore, it is less elastic compared with the adductor magnus tendon fixation. Monllau et al. reported excellent clinical and radiological outcomes in 16 adults who underwent isolated double-bundle MPFL reconstruction using the adductor magnus tendon sling technique. However, Gomes et al. and Monllau et al. included adult patients with different anatomical and biomechanical characteristics from children and adolescents. Yercan et al. reported no redislocations in three cases (four knees) following reconstruction using the sling technique. In contrast, Lind et al. reported 20% redislocation rates and 25% subluxation rates in 24 patients who underwent single-bundle MPFL reconstructions using the sling procedure. Notably, 5 of the 24 patients needed revision within one year of surgery due to recurrent patellar instability. Suturing the graft to the proximal (MCL) insertion area beside the adductor sling may result in graft elongation, which increases the risk of instability. In this study, we performed double-bundle MPFL reconstructions using a modified sling procedure whereby an isolated adductor magnus tendon sling was adopted at the femoral side. The procedure did not involve suturing or osteoperiosteal tunneling at the femoral side. It had the following advantages: (i) simple operation. (ii) fixation by looping against the adductor insertion maximized the quasi-isometric and elastic characteristics without causing over elongation of the graft. (iii) because no suturing of the adductor tendon or MCL was performed, the dynamic fixation allowed for rebalancing of the length and tension of the two bundles during postoperative flexion and extension. (iv) once a balance was achieved, the graft was immobilized and gradually healed together with the surrounding tissue. The MRI conducted postoperatively did not show any graft dysfunction (Fig. 4).

**Table 3:** Studies on the medial patellofemoral ligament (MPFL) reconstruction using the sling procedure

Authors	Study year	Population	Cases	Method	Follow-up period (months)	Graft	Complications	Ref
Yercan	2011	Children	4	Sling adductor magnus (AM) & suture; single-	15–20	Semitendinosus	None	[24]
Gomes	2008	Adult	12	bundle Sling AM insertion;	30–71	Semitendinosus	1 Subluxation	[25]
Monllau	2017	Adult	16	single-bundle Sling AM & suture;	Average 37.6	Gracilis	1 Aprehension 2 Flexion deficit	[9]
Lind	2016	Adolescent	17	Double-bundle	17–72	Gracilis	5 Redislocation; 5 Subluxation	[26]
this study	2022	Children and adolescent	21	Sling AM & medial collateral ligament suture; single-bundle Isolated sling; double-bundle	24–42	Anterior half peroneus lonGus tendon	None	

Adolescents have a smaller patellar volume and thus tunneling may increase the risk of patellar fractures. Therefore, in this study, patella perforation and graft fixation were performed using looped back braided sutures. In previous studies, the use of similar techniques was shown to be effective [21]. In this study, two 2.0 mm holes were drilled into the patella. Further, the bone bridge was maintained at 10 to 15 mm. This method significantly reduced the risk of patella fracture and improved patella growth. More importantly, the method allowed precise regulation of the reconstructed MPFL tension intraoperatively.

According to Zhao et al., the average failure load of the AHPLT was 322.35 ± 63.18 N [22], closing to the strength of the semitendinosus tendon, and the applicable length of AHPLT was about 24 cm. Besides, cutting only the anterior 1/2 to 2/3 of AHPLT maximizes structural integrity. The small incision point for harvesting the tendon is hidden behind the ankle joint, making it aesthetically appealing (Fig. 2b, c). Therefore, the AHPLT is an efficient autograft for the reconstruction of MPFL.

In arthroscopic surgery, only a small capsular window is required to expose the adductor and the medial patellar facet [23]. Furthermore, lateral retinacular release, debridement of small chondral fragments, and meniscus plasty or repair can be performed simultaneously. Arthroscopic surgeries are minimally invasive and more aesthetically appealing than open surgeries. Additionally, arthroscopic surgeries are more suitable for obese patients and patients who are overly distressed by scars. This study provides ideas for the improvement of MPFL reconstruction. Future large-scale studies are warranted to evaluate the long-term outcomes of the proposed technique.

## Conclusion

Arthroscopic reconstruction of the MPFL using a modified sling procedure is an effective procedure for the treatment of patellar instability in skeletally immature patients. This technique improved functional scores and radiological outcomes without major complications.

## Data Availability

The datasets generated and analysed during the current study are available from the corresponding author on reasonable request.
